# Physician and Patient Barriers to Breast Cancer Preventive Therapy

**DOI:** 10.1007/s12609-016-0216-5

**Published:** 2016-06-13

**Authors:** Susan Hum, Melinda Wu, Sandhya Pruthi, Ruth Heisey

**Affiliations:** 1 Department of Family & Community Medicine, Women’s College Hospital, 76 Grenville St, Toronto, ON M5S 1B2 Canada; 2 Department of Family & Community Medicine, University of Toronto, Women’s College Hospital, Princess Margaret Hospital, 76 Grenville St, Toronto, ON M5S 1B2 Canada; 3 Department of Medicine, Division of General Internal Medicine, Mayo Clinic, 200 First St SW, Rochester, MN 55905 USA

**Keywords:** Breast cancer, Chemoprevention, Preventive therapy, Decision-making, Selective estrogen receptor modulators, SERMs, Aromatase inhibitors, AIs, Uptake

## Abstract

The uptake of selective estrogen receptor modulators (SERMs) and aromatase inhibitors (AIs) for the primary prevention of breast cancer is low, despite their proven efficacy in several randomized clinical trials. This review summarizes the latest data on physicians’ and women’s barriers to breast cancer preventive therapy. Physicians’ challenges include: identifying suitable candidates for preventive therapy, inadequate training and confidence in risk assessment and counselling, insufficient knowledge of risk-reducing medications, and lack of time. High-risk women fear medication side effects, and they often weigh experiences of others more heavily than statistical probabilities to guide their decision-making. Despite decision aid interventions to help women make an informed decision, acceptance of preventive therapy will remain low until: risk/benefit profiles are more favorable, physicians are better educated and skilled in having these discussions, and suitable biomarkers to monitor drug efficacy and better clinical risk prediction models to assess true individual risk are available.

## Introduction

Two selective estrogen receptor modulators (SERMs), tamoxifen and raloxifene, are FDA-approved for the primary prevention of breast cancer—tamoxifen for both pre- and post-menopausal women, and raloxifene for post-menopausal women only [1•, 2]. Two aromatase inhibitors (AIs) that are not yet FDA-approved, exemestane and anastrozole, have been evaluated for breast cancer prevention in post-menopausal women only [1•, 3]. Although an estimated 15 % of high-risk American women aged 35–79 years are eligible for breast cancer preventive therapy [4], <5 % who are offered this option accept it [5]. Reasons for the low uptake of SERMs and AIs by high-risk women, and primary care physicians’ reluctance to recommend or prescribe it are multi-factorial. In this review, we will explore the latest data on both physicians’ and high-risk women’s barriers to the uptake of breast cancer preventive therapy. Future directions and strategies to improve uptake will be discussed using the ABC (Agents-Biomarkers-Cohorts) paradigm: effective non-toxic Agents, intermediary Biomarkers to assess drug efficacy, and better identification of high-risk Cohorts using clinical risk prediction models [6•].

## Risk-Reduction Therapies for Primary Prevention of Breast Cancer

SERMs and AIs have been evaluated for the primary prevention of breast cancer in women aged ≥35 years in several randomized clinical trials [1•]. Women aged ≥35 with a life expectancy of ≥10 years and a 5-year Gail risk score ≥1.66 % or a personal history of lobular carcinoma in-situ (LCIS) may be considered for risk-reducing pharmacotherapy.

The NSABP Breast Cancer Prevention Trial (BCPT P-1) randomized healthy pre- and post-menopausal women aged 35 years and older with a 5-year Gail model risk score of ≥1.66 % or a history of LCIS to receive tamoxifen 20 mg daily for 5 years versus placebo. Results demonstrated that treatment with tamoxifen significantly reduced the risk of invasive and noninvasive breast cancer by 49 and 50 %, respectively [7]. The reduction in breast cancer incidence was most pronounced in women with a history of LCIS (56 %) and atypical hyperplasia (AH) (86 %). Tamoxifen reduced the risk of ER-positive tumors, but showed no significant effect on ER-negative tumors. Significant toxicities experienced in the tamoxifen group were mostly stratified to women over the age of 50. These included hot flashes, invasive endometrial cancer (RR = 2.53; 95 % CI 1.35–4.97), and cataract formation (RR = 1.57). An increased incidence of thromboembolic events, including stroke (RR = 1.45; 95 % CI 0.93–2.77), deep vein thrombosis (RR = 1.60; 95 % CI 0.91–2.86), and pulmonary embolism (RR = 3.01; 95 % CI 1.15–9.27) was also observed, with higher risk associated with those aged >50 years [7]. In 1998, based on the results of the BCPT P-1 study, tamoxifen received FDA approval as a risk-reduction agent for women at increased risk of invasive breast cancer [1•].

Additional European studies have evaluated the effect of tamoxifen in women at average and increased risk of breast cancer. These studies demonstrated that tamoxifen reduced ER-positive breast cancers by about one third when compared to placebo [8–10].

Raloxifene is a second-generation SERM that was initially shown to be as effective as tamoxifen in reducing the risk of ER-positive breast cancers [11]. The Study of Tamoxifen and Raloxifene (STAR P-2) trial compared the two agents in healthy, high-risk post-menopausal women. The two treatment groups showed similar reduction in breast cancer incidence. Women treated with raloxifene experienced fewer thromboembolic events and cataracts and non-statistically significant lower rates of endometrial cancer. Updated results following the original report demonstrated that raloxifene was 76 % as effective as tamoxifen in reducing the overall risk of invasive disease (RR = 1.24; 95 % CI 1.05–1.47). In 2007, raloxifene was FDA-approved as a breast cancer risk-reduction agent [1•].

Two AIs, exemestane and anastrozole, have been evaluated for the primary prevention of breast cancer in post-menopausal women. In a randomized, placebo-controlled trial, exemestane significantly reduced invasive breast cancers (RR = 0.65; 95 % CI 0.18–0.70) without the toxicities (thromboembolic, cancer events) associated with SERM therapy [3]. Arthralgias and menopausal symptoms, including hot flashes, were the main adverse events in women taking exemestane, but women’s quality of life was minimally affected, although age-related bone loss worsened despite adequate intake of calcium and Vitamin D [12, 13].

Similar to the MAP.3 trial, the IBIS-II trial compared the AI anastrozole to placebo, demonstrating a significant decrease in risk of invasive breast cancer and a reduction in ER-positive tumors by 50 and 58 %, respectively [14]. Overall, data regarding the use of AIs as risk-reduction therapy for breast cancer are limited to post-menopausal women with a Gail risk score of >1.66 % or personal history of LCIS. The benefits and risks of AIs in comparison to tamoxifen and raloxifene have not been directly evaluated.

## Physicians’ Barriers

Despite the large number of women who qualify for breast cancer preventive therapy and the US Preventive Services Task Force’s recommendation [15] to counsel at-risk women aged ≥35 years (B recommendation) with a 5-year risk of >3 % where benefit outweighs risk, many are not offered this option [4]. A cross-sectional, web-based survey of physicians [16] revealed that only 13 % of providers had recommended or prescribed preventive therapy (8 % family medicine, 9 % internal medicine, 30 % gynecology).

A major barrier to discussing or prescribing preventive therapy is lack of confidence in identifying suitable high-risk patients. Primary care physicians (PCPs) are often the first contact person for a woman who wants to discuss her family history and concerns about preventing breast cancer, and PCPs consider it their responsibility to assess breast cancer risk [17, 18]. They are comfortable with taking a family history, yet often neglect to ask about reproductive history or history of abnormal breast biopsies [17]. Women with breast biopsies that impart a significantly increased risk of breast cancer (AH, LCIS) are more inclined to consider preventive therapy, yet they are not often identified as potential candidates [19]. Most PCPs have never used the Gail model [20], which is currently the best available tool to identify suitable candidates for preventive therapy [16, 17]. In a cross-sectional survey of 300 PCPs, only 37 and 33 % of internists and family physicians, respectively, reported ever using the Gail model, as compared to 60 % of gynecologists [16].

Other factors that prevent physicians from discussing preventive therapy with high-risk women include: lack of knowledge and insufficient training in risk counselling (i.e., oncologists are better trained) [17, 21], lack of knowledge of risk-reducing medication options [21], and concerns with medication side effects [22•].

Physicians are more comfortable advising about lifestyle modifications (e.g., weight reduction, exercise, and reducing alcohol intake), which according to observational studies may reduce breast cancer risk by up to 30 % [23•]. It is acknowledged that physicians’ recommendations are influential on women’s uptake of preventive screening maneuvers [24], and acceptance of risk-reduction therapies [25]. However, time constraints in a typical 15-min clinical encounter makes it challenging for front-line clinicians to take a thorough family history, a history of breast cancer risk factors, including previous abnormal biopsies and use risk-assessment tools, much less counsel about the risks and benefits of preventive therapy.

Other factors that steer physicians away from recommending preventive therapy include the lack of evidence of a decrease in breast cancer-specific and all-cause mortality [6•], concerns about the lack of validated intermediary biomarkers to monitor efficacy of treatment (unlike cardiovascular disease prevention with low-density lipoprotein (LDL) as a biomarker to guide statin use) [22•], and the fact that certain preventive therapies (AIs) are off-label [6•].

## Women’s Barriers

The acceptance of breast cancer risk-reduction therapy is low, with significantly higher uptake rates in clinical trials (25.2 %) than in non-trial settings (8.7 %; *p* < 0.0001) [26, 27]. Factors influencing women’s decision-making include: fear of side effects, knowledge and beliefs/concerns about preventive medications, confusion between chemotherapy and chemoprevention, cost, and lack of validated biomarkers to monitor drug efficacy.

### Fear of Side Effects

The most important barrier to accepting breast cancer preventive therapy by high-risk women is fear and concern about medication side effects. The most feared side effects are endometrial cancer, risk of blood clots and strokes (SERMS), and decrease in bone mineral density (AIs) [26–28]. Women are also concerned about more common adverse effects, such as hot flashes, other menopausal symptoms, and musculoskeletal and urogenital symptoms, which are medically “less serious,” but can negatively affect day-to-day activities and overall quality of life [29]. Some high-risk menopausal women are reluctant to give up hormonal replacement for preventive therapy [26]. Some women decline preventive therapy because they consider the risk of medication side effects more concerning than the risk of developing breast cancer [30, 31]. Decision aids or educational interventions advising women on the risks and benefits of preventive therapy may result in women becoming less inclined to start it [5, 32]. Even when women receive balanced risk/benefit profiles, their perception of developing medication side effects is over 40 % [6•, 27, 28].

### Knowledge and Beliefs About Preventive Therapy

Awareness of the availability of breast cancer preventive therapy is limited [33], even among a group of highly educated women [34]. Of those who are aware, many doubt the efficacy of SERMS or AIs in reducing breast cancer risk [27, 28, 32, 33]. In a Canadian study, high-risk women demonstrated interest in preventive therapy (62–67 % self-reported likelihood of taking it in the next 5 years), but required strong evidence of its efficacy and belief that side effects would be tolerable. They were more likely to take medication if they were “proactive” in health-related matters [30]. Women less likely to take preventive therapy were often strongly influenced by family members, relatives, or close friends who had experienced adverse side effects or developed recurrent breast cancer while taking tamoxifen as adjuvant therapy [27, 29].

### Preventive Therapy (Chemoprevention) Versus Chemotherapy

Chemoprevention is often confused with chemotherapy [26, 28, 30], especially since many women view tamoxifen as a “breast cancer drug” for recurrent disease [29]. Unsurprisingly, some women cited fear of side effects, such as hair loss, nausea, and vomiting, which are associated with chemotherapy [28, 29, 35].

### Concerns About Medications

A general aversion to medications is another important barrier to preventive therapy. Many women view medications as “unnatural” [30] and decline to take them unless they are deemed “necessary” for treatment [35], rather than for prevention [36]. The “HRT fiasco” has also been cited as a strong influencer against medications [30]. Some women are afraid of taking medications or swallowing pills [30], and over 90 % of high-risk women would choose topical tamoxifen (gel form) over oral pills, to avoid systemic effects [34]. Daily pill use for 5 years is also burdensome [34, 39], and may serve as a constant reminder of breast cancer risk [29, 35]. Older women with comorbidities are also concerned about the inconvenience of taking additional pills and the possibility of drug interactions with their other medications [33, 34].

### Socio-Demographics/Social Determinants of Health

Socio-demographic factors such as age, race, education, income or employment status, insurance status, and costs have not been associated with lower uptake rates [5, 26]. In earlier studies before Obamacare (Affordable Care Act), cost and health insurance coverage were important obstacles, especially for low-income women [33, 36]. Since September 2014, the Affordable Care Act has made tamoxifen/raloxifene risk-reduction therapy affordable since no copayment or deductible is required. However, its impact on uptake has yet to be determined [37].

## Other Barriers to Preventive Therapy

Some women report doubting risk calculation scores, because they worry that not all risk factors (such as breast density) are taken into account in these calculations [38]. Another barrier is the inability to prospectively identify women who might develop ER-positive versus ER-negative tumors [27]. The absence of validated intermediary biomarkers to assess response to medication as an indicator of risk-reduction [39], analogous to LDL levels to determine response to statins, is a significant barrier [22•, 27].

## Factors Associated With Higher Uptake

Women with a history of breast lesions imparting an increased breast cancer risk (atypical ductal hyperplasia or LCIS) [26] and with a Gail score >6 % are more likely to accept preventive therapy [27, 28]. Physicians’ recommendation for preventive therapy is influential in guiding women’s decision-making [25, 26, 30]. Women with a higher perceived personal risk of developing breast cancer [5, 26, 39], or higher anxiety around their perceived risk, are also more likely to accept the benefits of preventive therapy over the perceived risks [40]. A summary of factors associated with high-risk women’s uptake of breast cancer preventive therapy is presented in Table 1.

**Table 1:** Factors associated with uptake of breast cancer preventive therapy

Lower uptake	Higher uptake
Fear and concerns about side effects (both rare and medically less serious ones that can affect quality of life)	Abnormal biopsy results (e.g., women with atypical hyperplasia/LCIS vs. high-risk women from the general population)
Confusion between chemoprevention vs. chemotherapy	Higher perceived personal risk of breast cancer
Personal, family, or close friends’ experiences with breast cancer, chemoprevention, and chemotherapy	Fewer concerns about medication side effects and prevention trials
Greater knowledge of medication side effects	Physician recommendation
Cost/no health insurance coverage	
Uncertainty about breast cancer risk and risk-assessment models	
Daily pill burden	

## Discussion

An estimated 10 million American women are eligible for breast cancer chemoprevention [4], yet <5 % who are offered this option accept it [5, 22•]. Reasons for the low uptake of preventive therapy by high-risk women (see Table 1), and primary care physicians’ reluctance to recommend or prescribe it, are multi-factorial. The most common barrier to accepting preventive therapy is the fear of medication side effects, including life threatening but rare risks, such as thromboembolic events and endometrial cancer, and more common, but still infrequent ones, such as menopausal and musculoskeletal symptoms [26–29]. Acceptance or rejection of breast cancer preventive therapy by high-risk women is a “preference-sensitive decision,” [5, 35] and involves complex risk-benefit trade-offs [32, 35]. Qualitative studies revealed that high-risk women initially use all available information from their Gail risk score and risk/benefit profiles from decision aids or educational interventions to help in their decision-making, but their final choice is guided primarily by the experiences of significant others. Close family members and/or friends who have undergone breast biopsies, chemotherapy, or risk-reduction therapy impart experiences that ultimately shaped the attitudes and beliefs of high-risk women regarding breast cancer, personal risks, and medication use in general [35, 36].

Women are motivated to consider preventive therapy when they feel highly vulnerable, such as when they have physical signs and symptoms or abnormal mammographic findings [36]. High-risk women weigh the risk/benefits by evaluating how medication side effects might impact their daily lives, and which events (i.e., developing cancer or treatment side effects) would be experientially worse [5, 30, 35, 36]. Since women’s decision-making is not based exclusively on evidence-based findings [30, 35, 36], this partly explains why decision support interventions have had limited success in increasing preventive therapy uptake rates [22•]. This also explains why a strong patient-provider relationship may facilitate discussions about preventive therapy, when a PCP not only counsels using objective risk estimates but also takes into account a woman’s values and preferences in the shared decision-making process [41, 42].

PCPs’ difficulty in identifying suitable high-risk candidates has been cited as one barrier to discussing or prescribing breast cancer preventive therapy [27]. To address this gap, support tools for providers have been created [43•] with clearer messages about the ideal candidates for preventive therapy as demonstrated by the USPSTF chemoprevention consensus statement: cohorts of women with the highest benefit/risk ratio, such as those under 50 years of age, with a strong family history of breast cancer or a personal history of AH [44]. Physicians prefer electronically based, easy-to-use point of care tools, and the modified Gail or breast cancer risk-assessment tool (BCRAT) is one such tool [45]. It has been validated in white, Asian, and African American populations [46–48]. It is the best tool to identify candidates for preventive therapy, but is not effective at categorizing candidates for genetic testing or enhanced screening. The Breast Referral Screening Tool (B-RST) [49] and the Tyrer-Cuzick (IBIS) Tool [50] are better suited for such indications, respectively. These latter tools may be used to further stratify high-risk women, who could benefit from other risk-reduction strategies, such as lifestyle changes, prophylactic mastectomy, and/or prophylactic oophorectomy [22•, 23•, 51, 52].

Despite decision aid interventions using currently available risk calculators and discussions with PCPs about the risk/benefits of preventive therapy to help women make an informed decision, its acceptance will remain low, until medications have fewer side effects, biomarkers can be identified to monitor drug efficacy, and newer clinical risk prediction models can provide true individual risk versus population risk.

## Future Directions

Future directions and strategies to improve preventive therapy uptake requires attention to the ABC (Agents-Biomarkers-Cohorts) paradigm: the availability of effective non-toxic Agents, effective intermediary Biomarkers to assess response to medication, and better identification of high-risk Cohorts [6•].

New research on genome-wide association studies has led to more recent understanding of combined association of single nucleotide polymorphisms (SNPs) with increased risk for developing breast cancer. Incorporating SNP information into clinical risk prediction models has the ability to better inform individual risk, which in turn could improve uptake of preventive therapy going forward [53].

Overcoming a major obstacle to breast cancer pharmacotherapy necessitates improving its side effect profile. One strategy might be to either offer low- or intermittent doses of existing preventive agents, which would cause fewer adverse side effects [6•]. Clinical trials and observational studies are being investigated to test the efficacy and effects of varying doses of tamoxifen (1, 5, or 10 mg daily vs. 10–20 mg weekly vs. standard 20 mg daily dose) on systemic biomarkers, such as lipid and insulin-like growth factors, and tissue biomarkers, like Ki-67 [6•, 54]. Another approach is offering a topical form of tamoxifen (4-OHT) that can be applied directly onto the breast, and which is expected to decrease systemic effects [6•, 54]. Repurposed drugs, such as aspirin, metformin, NSAIDS, vitamin D, retinoids, statins, and bisphosphonates [6•, 41], are being investigated as potential preventive therapeutic agents with better safety profiles than SERMs and AIs, and possible effectiveness against ER-negative tumors as well [51, 55].

The lack of surrogate biomarkers is another obstacle to the uptake of preventive therapy by high-risk women. Mammographic density has been identified as a risk factor for breast cancer, and it is now being investigated as a biomarker of response to breast cancer chemoprevention [22•, 41]. Enrollment in the IBIS-I trial caused a significant decrease (*p* < 0.001) in mammographic density after 18 months on tamoxifen, as compared to placebo [56], with a 10 % decrease in mammographic density associated with a 63 % reduction in breast cancer risk [57].

To realistically decrease breast cancer incidence rates, non-pharmacologic interventions such as lifestyle modifications, which may reduce breast cancer risk by 30 %, should be offered alongside chemoprevention [22•, 23•, 51, 52]. Excess weight has been linked to the development of both ER-positive and ER-negative tumors, and a 5–10 % weight loss in the years before and shortly after menopause has been associated with a 25–50 % reduction in breast cancer risk [51]. These lifestyle recommendations for breast cancer risk-reduction also reduce the incidence of diabetes, hypertension, heart disease, and dementia [22•, 23•, 51, 52]. PCPs are well positioned to counsel and encourage healthy lifestyle changes, so that breast cancer risk and concurrent co-morbid conditions can be addressed simultaneously. High-risk women are largely receptive to breast cancer screening [24], and with the future availability of biomarkers and less toxic medications, preventive therapy may become more widely accepted.

## Conclusions

The uptake of SERMs and AIs for the primary prevention of breast cancer is low, despite its proven efficacy in randomized clinical trials over the past decade. PCPs have difficulty identifying suitable candidates, lack knowledge about therapeutic options and confidence to counsel about preventive therapy. High-risk women’s decision to accept chemoprevention is complex, and their choice often reflects lived experiences, rather than statistical probabilities. Despite decision aid interventions to help women make an informed choice about preventive therapy, acceptance will remain low until physicians are better educated and more skilled in having these discussions, medications are safer and better tolerated, suitable biomarkers to monitor drug efficacy are identified, and clinical risk prediction models using genomic information can provide true individual risk. Risk/benefit counselling about preventive therapy, alongside lifestyle modifications to high-risk women, will simultaneously address breast cancer risk-reduction, and the prevention of co-morbidities, such as diabetes and cardiovascular disease.
